# Modularity and Intrinsic Evolvability of Hsp90-Buffered Change

**DOI:** 10.1371/journal.pone.0000076

**Published:** 2006-12-20

**Authors:** Charles C. Carey, Kristen F. Gorman, Suzannah Rutherford

**Affiliations:** Division of Basic Sciences, Fred Hutchinson Cancer Research Center Seattle, Washington, United States of America; Max Planck Institute of Molecular Cell Biology and Genetics, Germany

## Abstract

Hsp90 controls dramatic phenotypic transitions in a wide array of morphological features of many organisms. The genetic-background dependence of specific abnormalities and their response to laboratory selection suggested Hsp90 could be an ‘evolutionary capacitor’, allowing developmental variation to accumulate as neutral alleles under normal conditions and manifest selectable morphological differences during environmental stress. The relevance of Hsp90-buffered variation for evolution has been most often challenged by the idea that large morphological changes controlled by Hsp90 are unconditionally deleterious. To address this issue, we tested an Hsp90-buffered abnormality in Drosophila for unselected pleiotropic effects and correlated fitness costs. Up to 120-fold differences in penetrance among six highly related selection lines, started from an initially small number of flies and rapidly selected for and against a deformed eye trait (*dfe*), did not translate into measurable differences in any of several tests of viability, lifespan or competitive fitness. Nor were 17 *dfe* Quantitative Trait Loci (QTL) associated with fitness effects in over 1,400 recombinant lines. Our ability to detect measurable effects of inbreeding, media environment and the *white* mutation in the selection line backgrounds independent of *dfe* penetrance suggests that, within the limitations of laboratory tests of fitness, this large morphological change controlled by Hsp90 was selectable independent of strong, correlated and unconditionally deleterious effects—abundant, polygenic variation hidden by Hsp90 allows potentially deleterious alleles to be readily replaced during selection by less deleterious alleles with similar phenotypic effects. Hsp90 links environmental stress with the expression of developmental variation controlling unprecedented morphological plasticity. As outlined here and in the companion paper of this issue, the complex genetic architecture of Hsp90-buffered variation supports a remarkable modularity of Hsp90 effects on quantitative and qualitative phenotypes, consistent with the ‘Hsp90 capacitor hypothesis’ and standard quantitative genetic models of threshold traits.

## Introduction

Across diverse species, the Hsp90 chaperone buffers widespread morphological variation and controls characteristic developmental transitions, for example altered morphologies in laboratory and wild populations of Drosophila [1] and Arabidopsis [2], cortical patterning in Tetrahymena [3], [4], developmental progression in fungi [5], [6], and metamorphosis in Leishmania parasites [7] and Ascidian embryos [8]. Depending on genetic background, Hsp90 controls nearly any morphological feature of adult flies [1] or Arabidopsis seedlings [2]. The expression of Hsp90 buffered phenotypes in Drosophila depends on the chance segregation of multiple, previously-undetected polymorphisms [1] (and unpublished results). In nature, a balance between Hsp90 buffering and the expression of morphological traits is likely controlled by environmental stress. Stress-damaged proteins titrate Hsp90 from its signal transduction targets. Despite induction of Hsp90 and other chaperones by the heat shock response, it is believed that severe stresses can temporarily overwhelm the Hsp90 chaperone system [9]. Abnormal phenotypes that are controlled by Hsp90 occur during laboratory heat stress [3], [4] and by associated chaperones during stress in nature [10], but the relevance of Hsp90-buffered variation for evolution is challenged by the idea that any potentially adaptive morphological variations controlled by Hsp90 have necessarily pleiotropic and unconditionally deleterious side-effects [2], [11]–[14].

In the companion paper of this issue [15], we showed that Hsp90 controls the genetic variability and predicted selection responses (‘extrinsic evolvability’) of highly discrete (or canalized) quantitative traits. However, the rare likelihood that any particular change in a previously optimized and invariant phenotype could increase fitness or performance also depends on the traits ‘intrinsic evolvability’; genetic and developmental modularity enabling changes to occur independent of pleiotropic effects (unselected changes in traits unrelated to the trait under selection and correlated fitness costs). If Hsp90-buffered changes in Drosophila morphology were unconditionally deleterious, they could not be selected in nature, and would not contribute to adaptation under any circumstance. However, in the accompanying paper we show that quantitative variation in symmetry between left and right wing areas, almost certainly deleterious in any environment imaginable, was tightly buffered independent of Hsp90, even under low Hsp90 conditions where variation in previously-invariant bristle traits was dramatically increased [15]. The qualitative phenotypes described previously [1], [16] resemble large developmental mutations often associated with pleiotropic side-effects and inescapably reduced fitness (“hopeless monsters”; [17]), but they are controlled by Hsp90 and the concerted action of multiple alleles in many genes. Here we provide evidence that qualitative changes buffered by Hsp90 are selectable independent of large and unconditionally deleterious effects, a conclusion logically supported by threshold trait mechanisms.

## Results

Hsp90-buffered variation in Drosophila is distinguished by the sheer number and variety of adult morphologies controlled by the interaction of Hsp90 with hidden variation specific to different strain backgrounds [15], [16], [18]. Remarkably, even the most extreme abnormalities are in general readily transmitted to the progeny of affected flies and enriched to high frequency by artificial selection [1]. To determine whether morphological plasticity controlled by Hsp90 could evolve independent of large deleterious fitness effects, we measured correlated fitness costs of lines artificially selected for an eye deformity controlled by Hsp90 (*dfe*). High lines (*HE1, HE2, HE3*), expressing *dfe* at high frequency, and genetically-related low lines (*LE1, LE2, LE3*), selected against *dfe* and expressing deformities only occasionally, were started from just a single deformed male and 3–4 unaffected females of the same genotype [1]. Penetrance increased in their progeny and subsequent generations to the point *dfe* was expressed at high frequency, even after the Hsp90 mutation that originally revealed the trait was lost due to almost certain selection against flies carrying Hsp90 mutations. Crossing Hsp90 mutations back into *dfe* lines further enhanced *dfe* penetrance, demonstrating that Hsp90, while neither necessary nor sufficient for expression of *dfe*, continued to buffer cryptic variation for the trait.

### Absolute fitness: viability, survival and longevity

Major developmental mutations and large morphological changes often have pleiotropic effects and serious fitness costs. The eyes of *dfe* flies range from defects as mild as duplications of bristles at the eye margin and reduced size, to protuberances, more severe bristle duplications and deformity. We do not think the specific changes in eye shape of *dfe* lines would be adaptive in any novel environment imaginable. However, to address whether a rare beneficial change controlled by Hsp90 could have the potential to evolve free of deleterious fitness effects, we compared the performance of high versus low penetrance *dfe* lines in several tests of fitness. Importantly, all *dfe* flies were mutant for the *white* gene (*w^1118^*), which encodes a general transporter that is required for eye pigmentation and normal vision [19]. Because of visual defects and other pleiotropic effects, *w^1118^* mutants have reduced competitive fitness [20]. For comparisons of fitness between the high and low lines, all flies would have had similar visual defects, independent of the degree or penetrance of their deformity.

**Figure 1:**
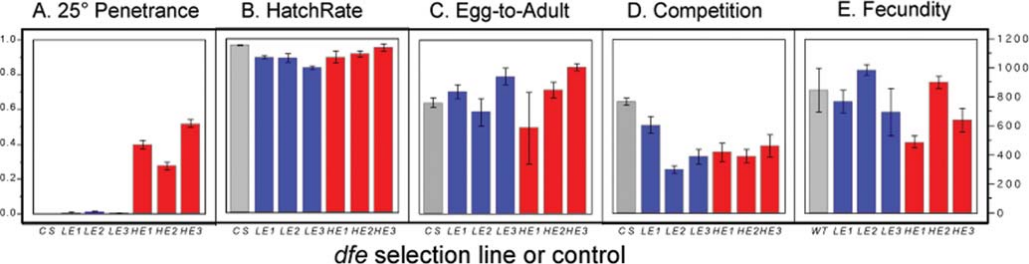
Representative tests of penetrance, absolute and relative fitness. High (red) and low (blue) lines, genetically differentiated for *dfe* penetrance were not differentiated by tests of viability and fitness. Controls (grey) were either *Canton-S* (*CS*) flies tested at the same time or published values for wild-type flies (WT; [21]). Experiments showing the most extreme between line differences are displayed. Error bars indicate the standard errors of the mean. **A.** Penetrance of eye deformity in *dfe* lines and *CS* flies reared at 25°C. **B.** Hatch rate of timed embryo collections measured at 36 hours post egg-laying. **C.** Egg-to-adult survival measured as the number of adult flies emerging versus eggs laid. **D.** Fractional survivial of *dfe* progeny in competition with *CS* flies co-cultured at high density. **E.** Lifetime female production of eggs (wild-type values; [21]) or viable adult offspring (high and low line *dfe* females).

After 42 generations of selection, the high lines were further inbred to increase *dfe* penetrance, reduce genetic variation and enrich *dfe* polymorphisms (see methods). We first tested the inbred *dfe* high lines and related low lines for effects of the deformity on embryonic viability and egg to adult survival. The penetrance of high (*HE1*–3) and low (*LE1*–3) lines was compared with mean measures of absolute fitness and competitive fitness relative to wild-type flies. As shown in Figure 1, high (red) and low lines (blue) were clearly differentiated for *dfe* penetrance, at 25°C (panel A), but did not differ in any measure of fitness. Significant line effects were not systematically distributed to high or low, and were more likely attributable to the random effects of inbreeding and genetic drift than to fitness effects of the deformity (Table 1). Perhaps surprisingly, given the degree to which head structures are affected in *dfe* flies, embryonic development and egg to adult survival were similar to that of wild-type flies (Fig. 1, panels B and C). Indeed, for any simple test of absolute fitness, there was no relationship between penetrance and viability, and no measurable fitness differences between high and low selection lines or between *dfe* selection lines and wild-type, *Canton-S* (*CS*) controls. While it is widely expected that such extreme deformities should decrease the viability of affected flies, this has not been our experience in breeding Hsp90 buffered abnormalities generally [1], and no viability effects specific to the *dfe* trait were detected here.

**Table 1:**
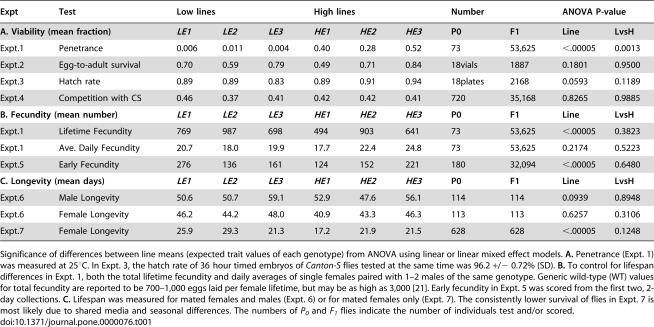
Mean line effects and effects of low versus high (LvsH) penetrance in several tests of *dfe* fitness.

Expt	Test	Low lines			High lines			Number		ANOVA P-value	
**A. Viability (mean fraction)**		***LE1***	***LE2***	***LE3***	***HE1***	***HE2***	***HE3***	**P0**	**F1**	**Line**	**LvsH**
Expt.1	Penetrance	0.006	0.011	0.004	0.40	0.28	0.52	73	53,625	<.00005	0.0013
Expt.2	Egg-to-adult survival	0.70	0.59	0.79	0.49	0.71	0.84	18vials	1887	0.1801	0.9500
Expt.3	Hatch rate	0.89	0.89	0.83	0.89	0.91	0.94	18plates	2168	0.0593	0.1189
Expt.4	Competition with CS	0.46	0.37	0.41	0.42	0.42	0.41	720	35,168	0.8265	0.9885
**B. Fecundity (mean number)**		***LE1***	***LE2***	***LE3***	***HE1***	***HE2***	***HE3***	**P0**	**F1**	**Line**	**LvsH**
Expt.1	Lifetime Fecundity	769	987	698	494	903	641	73	53,625	<.00005	0.3823
Expt.1	Ave. Daily Fecundity	20.7	18.0	19.9	17.7	22.4	24.8	73	53,625	0.2174	0.5223
Expt.5	Early Fecundity	276	136	161	124	152	221	180	32,094	<.00005	0.6480
**C. Longevity (mean days)**		***LE1***	***LE2***	***LE3***	***HE1***	***HE2***	***HE3***	**P0**	**F1**	**Line**	**LvsH**
Expt.6	Male Longevity	50.6	50.7	59.1	52.9	47.6	56.1	114	114	0.0939	0.8948
Expt.6	Female Longevity	46.2	44.2	48.0	40.9	43.3	46.3	113	113	0.6257	0.3106
Expt.7	Female Longevity	25.9	29.3	21.3	17.2	21.9	21.5	628	628	<.00005	0.1248

Significance of differences between line means (expected trait values of each genotype) from ANOVA using linear or linear mixed effect models. **A.** Penetrance (Expt. 1) was measured at 25°C. In Expt. 3, the hatch rate of 36 hour timed embryos of *Canton-S* flies tested at the same time was 96.2 +/− 0.72% (SD). **B.** To control for lifespan differences in Expt. 1, both the total lifetime fecundity and daily averages of single females paired with 1–2 males of the same genotype. Generic wild-type (WT) values for total fecundity are reported to be 700–1,000 eggs laid per female lifetime, but may be as high as 3,000 [21]. Early fecundity in Expt. 5 was scored from the first two, 2-day collections. **C.** Lifespan was measured for mated females and males (Expt. 6) or for mated females only (Expt. 7). The consistently lower survival of flies in Expt. 7 is most likely due to shared media and seasonal differences. The numbers of *P_0_* and *F_1_* flies indicate the number of individuals test and/or scored.

### Relative fitness: competition with wild-type

Tests of absolute fitness such as those described above could have missed fitness effects that depend on the frequency and fitness of more competitive genotypes. Therefore we next tested the fitness of the *dfe* selection lines relative to wild-type flies. Each of the white-eyed *dfe* lines was co-cultured in competition with *Canton-S* (*CS*; red eyed) flies. In crowded vials, wild-type flies out-competed any of the *dfe* lines, as expected due to potential inbreeding of *dfe* lines during selection and known deleterious fitness effects associated with the *w^1118^* mutation. In the most extreme case (the second transfer of Expt. 4; shown in Fig. 1D), line *LE1* had significantly higher relative fitness than any other *dfe* line, and was equally competitive with wild-type (50% total surviving flies were LE1 and 50% were CS). Even so, differences among the *dfe* lines did not translate into an overall difference between the high and low lines, and overall just 36% of surviving flies were from the *dfe* lines. When additional data were pooled over different rearing conditions and several transfers, including some 35,000 F_1_ flies in the study, the high or the low lines did not differ, and did equally poorly against wild-type (Expt. 4, Table 1). The overall differences between selection lines and wild-type in these experiments show that we were able to detect fitness effects of the *w^1118^* mutation and/or inbreeding of the selection line backgrounds. Our inability to detect a difference between the high and low lines suggests that neither the eye deformity itself nor the genetic variation producing the deformity had strongly deleterious effects.

### Female fecundity and longevity

**Figure 2:**
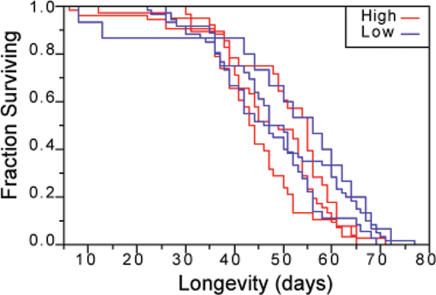
Survival curves for high and low *dfe* lines. For simplicity, male and female values were grouped and equal numbers of each sex within each line are represented. Multiple independent comparisons failed to detect longevity differences between high and low lines (Table 1).

To test for pleiotropic effects of *dfe* alleles, we scored the lifetime production of viable adult progeny of female flies. Fecundity was measured in aged cohorts of female and male flies reared under standard conditions and transferred at ∼two day intervals throughout their fertile life spans. Overall, female fecundity of *dfe* flies was similar to that reported for wild-type ([21]; Fig. 1E and Table 1B). The differences between lines disappeared when normalized for female lifespan, and additional tests of either lifespan (Table 1C and Fig. 2), or normalized daily fecundity (Table 1B) did not reveal differences between lines or between high and low penetrance. We did measure previously noted longevity differences between male and female flies under conditions where neither line nor line-by-sex effects were significant, and in a separate experiment, across the board sensitivity of *dfe* lines to differences in media and growth conditions were detected (Table 1; Expt.7). Despite a large number of independent experiments and flies tested, which did discriminate fitness effects due to environmental and genetic background effects, we found no measurable or systematic differences in fitness between the high and low lines, even when transfers and tests with the most extreme line differences were compared (Fig. 1).

### Genetic architecture of *dfe* selected independent of pleiotropic effects

**Figure 3:**
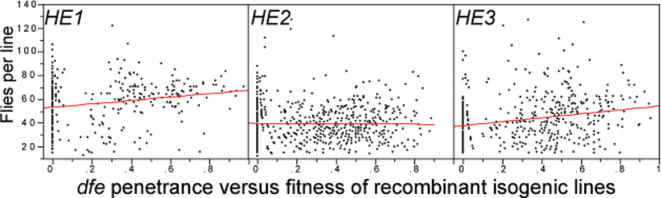
Regression of early fecundity and viability onto penetrance in 1,432 recombinant isogenic lines. If genes for *dfe* had effects on viability and survival, a negative relationship would be expected, however penetrance was positively correlated with fitness in recombinant lines from *HE1* and *HE3*.

To specifically examine the fitness effects of the genes controlling *dfe* we created 1,432 recombinant isogenic families (clones) containing a small portion of the extensively inbred, and highly homozygous *Samarkand* (*Sam*) background in the context of the second and third chromosomes from each inbred high line. Reasoning that recombinant families with greater penetrance carry more *dfe* alleles, in Figure 3 we asked whether viability and fecundity were inversely correlated with penetrance across the sets of recombinant lines. The relationship between fitness (viability and early fecundity) and penetrance of any *dfe* line was either not significant (*HE2*) or was even slightly positive (*HE1* and *HE3* ; in each case P<0.0005), possibly indicating that alleles from the unrelated and extremely inbred genotype *Sam*, enriched in the recombinant families with lower penetrance, were deleterious in the context of more fit, heterozygous high line backgrounds (*HE2/HE1* and *HE2/HE3*—see methods). Furthermore, among 17 *dfe* quantitative trait loci (QTL) mapped at 5–10 cM resolution across the three high lines, no *dfe* QTL consistently co-localized with a fecundity QTL or was associated with fitness effects when tested at closely linked markers (CC and SR, unpublished). In summary, while highly significant and expected effects of high versus low were measured for *dfe* penetrance, the high and low penetrance *dfe* lines did not differ in any of eight laboratory tests of fitness, countering widespread suggestions that Hsp90 buffered phenotypes in Drosophila necessarily suffer large, “unconditionally deleterious” fitness effect, the most frequent challenge to the ‘Hsp90 capacitor hypothesis’ [2], [11]–[14].

## Discussion

To our surprise, we found that Hsp90 buffering is remarkably specific to threshold traits, qualitative changes in morphology like *dfe* and the most invariant quantitative traits [15]. For thresholds resulting from non-linearity in developmental responses, small and often random changes in genetic and environmental factors can have large effects on penetrance when individuals or inbred populations are near the threshold, whereas away from trait thresholds even large effects are buffered. Our data suggest that multiple cryptic alleles capable of affecting Hsp90 buffered traits segregate in populations at high frequencies [1], [16]. In populations near a threshold for deformity like the *dfe* selection lines, abundant genes with small effects can interchangeably contribute to liability for the trait (probability of deformity). We suggest that the evolutionary dynamics of threshold traits under selection is qualitatively different from the evolution of continuous traits. At high frequency, conditionally cryptic alleles contribute interchangeably to threshold trait phenotypes, allowing different individuals in a highly affected population to carry different combinations of alleles and reducing inbreeding. The sheer abundance of trait polymorphisms would allow natural selection to eliminate polymorphisms with deleterious pleiotropic effects. For example, Hsp90 mutations themselves are lost coincident with artificial selection for deformity [1]. Over the course of selection, deleterious alleles can be replaced by less-deleterious alleles with similar phenotypic effects.

Despite the central importance of adaptive evolution to understanding the evolution and emergence of biological diversity, the time and average number of mutations required for a novel adaptation to appear, and the expected size of allelic effects remain largely unresolved. Under the assumptions of gradualist models of evolutionary adaptation, the mapping of phenotype onto fitness is smooth and monotonic, with a single peak of maximum fitness. On such a fitness landscape, the changes that are most likely to be favourable are slow, small and quantitative, rather than rapid, large and qualitative [22], [23]. Under alternative assumptions pioneered by Wright [24], adaptations can be large on a rugged fitness landscape with many local optima for fitness. The sudden production of novel adaptations independent of correlated negative fitness effects would facilitate “peak shifts” from previously adapted optimal phenotypes to distant phenotypic optima, effectively by-passing lower fitness intermediates. By allowing simultaneous selection of multiple alleles of small effect contributing to large phenotypic transitions at trait thresholds, ‘evolutionary capacitors’ such as Hsp90 may fulfil the predictions of both of these historically dichotomous models of evolutionary change.

### Hsp90 and evolvability

It has been suggested that many genes are ‘evolutionary capacitors’ [25], but several features of Hsp90 buffering distinguish it from well-documented genetic buffering effects (epistasis) of other known mutations [26]. First, Hsp90 activity is stress-sensitive and reversible. In order for most genes to act as an evolutionary capacitor, new potentially-adaptive ‘capacitor mutations’ would initially occur in a single, rare individual. In combination with previously cryptic alleles, this mutation must then provide a large enough fitness advantage to spread into the population without being lost [27]. By contrast, during severe environmental stress, reversible reductions of Hsp90 occur simultaneously in every individual of a population, effectively shifting the population as a whole to lower levels of developmental signaling. Many individuals with low levels of developmental signaling would cross thresholds, immediately exposing previously buffered alleles to selection. Second, while most populations maintain a large reservoir of quantitative trait variation, allowing virtually any normally-variable quantitative trait to be rapidly and effectively altered by selection, Hsp90 buffering favors typically-invariant discrete (or canalized) traits, which normally have low variability and a correspondingly low capacity to evolve. If an occasional Hsp90-buffered change were by chance beneficial in a novel and stressful environment, previously canalized traits have a rare opportunity to evolve. If not, the conditionally deleterious alleles exposed by Hsp90 would be removed by purifying selection. In the case of threshold traits only those individuals already at the extremes of signaling distributions are affected, even during severe stress [28]. Finally, the phenotypic effects of most mutations are restricted to specific pathways and/or processes. By contrast, Hsp90 is a chaperone for over 150 known targets and pathways (www.picard.ch/downloads/downloads.htm). These Hsp90 targets are key proteins in cell cycle, protein synthesis, gene regulatory, chromatin remodeling, growth control and morphogenesis networks between which Hsp90 is a hub [29].

Depending on genetic background, Hsp90 buffering can affect virtually any morphological feature of a presumably large range of organisms, extending from adult flies [1] to Arabidopsis seedlings [2]. We showed previously that selection enriches previously-buffered alleles to the point they become independent of Hsp90. Our new results suggest that adaptive Hsp90-buffered phenotypes, once exposed, are selectable independent of correlated fitness costs and unselected changes in other traits. Whether Hsp90 control of variability and canalization evolved as an evolutionary adaptation to unpredictable environmental changes or emerges as an unselected by-product of more immediate and vital cellular roles of the Hsp90 chaperone complex, its dual roles as a biochemical buffer of mis-folded proteins and a phenotypic buffer of diverse and unexpected morphology uniquely position the Hsp90 chaperone system to control evolvability and promote diversification and survival of stressed populations under pressure to adapt in novel environments.

## Materials and Methods

### Selection and inbreeding of *dfe* lines

The *deformed eye* selection was founded from F_1_ progeny of a cross between a heterozygous Hsp90 mutant (*19F2*; [1]) and another, historically related laboratory strain (both initially from the *w^1118^* background). A single, heterozygous *F_1_* male with an eye deformity was crossed to 3–4 related heterozygous siblings, yielding several more flies with eye deformities in the *F_2_* generation. By the *F_4_* generation, sufficient numbers of dfe flies were available to found six related, but independently maintained selection lines (3 high and 3 low eye penetrance lines *HE1*–3 and *LE1*–3 [1]). In subsequent generations, *dfe* flies were selected and crossed within each of the high lines, and a similar number of related normal sibs were selected and crossed within each of the low lines. With successive generations, *dfe* penetrance (fraction affected progeny) increased among the high lines to the point that sufficient *dfe* polymorphisms were present that the trait no longer depended on the Hsp90 mutation, which is apparently deleterious even in the heterozygous state. By generation 15, none of the flies from either high or low lines carried the Hsp90 mutation [1]. Fitness tests were initiated using inbred lines isolated from generation *F_42_* of selection. At generation *F_42_*, we scored the offspring of each of 10 to 12 single mated females reared at 29.5°C, selected at random from affected flies in each high line. The next generation, 10 to 12 females were again chosen from the vial with the highest penetrance within each line, and this process was repeated for several generations. This ‘isofemale’ selection was essentially based on breeding values, rather than selection at the level of individual *dfe* flies. High lines *HE2* and *HE3* responded to several rounds of selection and are likely the most inbred; the mean penetrance of dfe increased among the vials, and the coefficient of variation decreased 4–5 fold. Line *HE1* showed little response to this selection and a single isofemale line was chosen from the first round of selection. At 29° C high line penetrance and coefficients of variation were 0.43 (0.26), 0.73 (0.14) and 0.85 (0.07) for the inbred *HE1, HE2* and *HE3* lines respectively. During the inbreeding, artificial selection of the low lines was continued as usual without further inbreeding.

### Embryonic viability

The hatching success of embryos collected from each high and low *dfe* selection line and a wild-type control (*Canton-S*) was measured at 25°C and 60% humidity. Three 2–6 hour collections from independent groups of approximately 100 parents each were scored for each line and control. Each collection consisted of 100–140 embryos. Periodically, hatched larvae were removed from the collection plates. At 36 hours post-collection, empty and non-empty (unhatched) chorions were easily counted under a dissecting microscope. No further hatching was observed beyond 36 hours.

### Egg-to-adult survival

Embryos laid in vials on standard cornmeal molasses agar during a 48-hour collection (averaging 105 embryos/vial) were counted, and then allowed to develop to adulthood at 25°C at 60% humidity. Eclosing flies were scored at days 12, 15 and 17. Egg-to-adult survival was determined for each of 3 vials for each genotype as the ratio of embryos emerging as adult flies.

### Competition with wild-type

Relative fitness of low and high *dfe* lines in competition with wild-type flies was measured at a high density of competing flies (adjusted to less than 25% expected embryo-to-adult survival). Six 2–4 day old gravid females from each *dfe* line and six similarly-aged, non-virgin *Canton-S* competing females were placed in a vial for egg laying, with 10 vials (groups of flies) started for each genotype. To assure that the relative competitive of the parental females in this experiment were due to genetic rather than environmentally induced maternal, paternal or grandparent effects, all parental flies were simultaneously raised under equivalent density, sex ratio and age conditions. Every two days, the flies were transferred to new vials, for a total of four replicate vials per group of flies. The number of red (*Canton-S*) and white-eyed (*dfe*) adults emerging from the competition between *dfe* and wild-type larvae and parents was determined by scoring the vials on days 11,15, and 17. The ratio of white/total flies was used to measure relative fitness.

### Female fecundity

Lifetime and early female fecundity were scored at 25°C and 60% humidity and measured as the number of viable adult offspring produced. Therefore, this measure included number of eggs/laid per female and their offsprings' viability. To control for differences in fertile lifespan in Experiment 1, both total lifetime fecundity (Fig. 1E) and daily averages were recorded. In Experiment 5, we scored early fecundity (first 2 weeks) for single females paired with 2 males.

### Longevity

Longevity was measured as days from eclosion through death in mated males and females housed singly up through four flies per vial and transferred to fresh, yeasted vials with every 2–5 days. Male and female longevity in Experiment 6 was measured for approximately 20 flies per line. Female longevity in Experiment 7 was measured for 103–107 flies per line.

### *dfe* penetrance

Penetrance at 25°C was scored in male and female progeny of flies tested for lifetime fecundity in Experiment 1. 53,625 progeny of 1,431 recombinant lines (Fig. 3) were raised at 29°C and scored on days 10, 13 and 15. All experiments were humidity controlled (60%). Linear regressions were plotted and analyzed using JMP software (SAS Institute, Raleigh, NC). The effects of line, and low versus high groups of lines (LvsH) were determined using linear models (line) or linear mixed effects models (LvsH; treating line as a random effect) in R (www.r-project.org). For ratios near 0 or 1, data were arcsin transformed to enable better statistical discrimination of differences between individuals and lines. Lifespan fit the expectations of gaussian distributions, therefore no transformation of lifespan data was necessary, however Chi-square significance values of log rank or Wilcoxon tests of survival were also determined and gave similar results (JMP; SAS Institute Raleigh, NC).

### Construction of recombinant isogenic families

Small recombinant fragments of the highly uniform wild-type background, *Samarkand I–236* (*Sam*) were introduced into the *HE1, HE2* and *HE3* selection line backgrounds by 2 generations of backcrossing (decreasing the average fraction of *Sam* to 12.5%). To increase the number and variety of meiotic breakpoints and the genetic map distance, subsequent generations of free meiotic recombination were performed. At generation B_2_, four sublines were started within each line and carried through 5 more generations with round-robin mating. The resulting recombinant isogenic families (i.e. genetic “clones”) contained unique pairs of recombinant autosomes, isolated in males using the attached-autosome balancer *T(2∶3)B3* (Bloomington stock 2506) [30]. Each unique recombinant/balancer male was backcrossed to *HE2* females and their genetically identical progeny were first scored for *dfe* penetrance and then stored at −80°C for genotyping. In *HE2* recombinant families, the genotype of each marker was either *HE2/HE2* or *HE2/Sam*, but in the other recombinant families, the genotypes were heterozygous between *HE2* and each high line. Within the groups of recombinant families for any selection line, approximately 87.5% of the markers were expected to be high/high and 12.5% were expected to be high/*Sam*. Selected recombinant isogenic families were later genotyped at molecularly mapped STS sites (Drosophila genome project; [30]).
